# How to dissect the pelvic nerves: from microanatomy to surgical rules. An evidence-based clinical review

**DOI:** 10.52054/FVVO.14.1.011

**Published:** 2022-04-03

**Authors:** A Aleksandrov, A.V. Smith, R Botchorishvili, B Rabischong

**Affiliations:** Department of Gynecological Surgery, University Hospital Estaing, Clermont-Ferrand, France; Department of Obstetrics and Gynecology, Specialised Hospital for Obstetrics and Gynecology SBAGAL Pr. Dimitar Stamatov Varna, Medical University Varna, Bulgaria; Department of Obstetrics and Gynecology, Higueras Hospital, Concepcion, Chile.

**Keywords:** Pelvic nerves, anatomy, nerve sparing, rules of dissection

## Abstract

**Background:**

Advanced gynaecological procedures often include extensive pelvic dissections, with the nervous structures involved in the disease. Nerve-sparing and preservation is a key factor in reducing postoperative morbidity.

**Objectives:**

The goal of this review is to describe in detail the structure of the pelvic nerves and to gather information from other surgical specialties to give recommendations for safe nerve dissection applied in different gynaecological subspecialties.

**Materials and methods:**

An extensive literature review was carried out in PubMed and Google Scholar. The search included articles concerning peripheral nerve anatomy, mechanisms of injury and different dissection techniques, with the most exhaustive being analysed for the review. Articles from different fields of medicine like orthopaedics, plastic surgery, maxillofacial surgery dealing with peripheral nerve injuries and repair have been reviewed.

**Results:**

The following review demonstrates the in-depth anatomy and mechanism of injury of the peripheral nerves, describes the different techniques for neurolysis and proposes some directions for safe nerve dissection.

**Conclusion:**

When performing complex gynaecological surgeries, the surgeon should avoid unnecessary nerve handling, apply nerve-sparing techniques whenever possible and use the new devices to preserve the nervous structures. Advanced gynaecological surgeries should be performed in specialised centres by expert surgeons with comprehensive knowledge in neuropelveology.

**What is new?:**

To our knowledge, this is the first article focused on peripheral nerves that collects data from such a wide range of specialties in order to propose the most comprehensive recommendations that could be applied in pelvic surgery.

## Introduction

The peripheral nerves in the female pelvis are frequently encountered during advanced gynaecological procedures. Surgeries like promontofixation for pelvic organ prolapse, lymphadenectomy in the oncological cases or the treatment of deep endometriosis with nerve involvement could lead to severe postoperative morbidity and important deficits. In recent years, the concept of “nerve sparing” surgery has been widely incorporated in the different fields of gynaecology (Muallem et al., 2019; Ceccaroni et al., 2020; Zakhari et al., 2020; Darwish and Roman, 2017; Shiozawa et al., 2010; Ercoli et al., 2017). The nerve sparing techniques have shown excellent results in terms of decreased morbidity and shorter functional recovery after surgery compared to the standard techniques (De Resende et al., 2017; Gil-Moreno et al., 2019; Wu et al., 2019). The advance of minimally invasive surgery has additionally reinforced the development of nerve sparing, as the magnification of the endoscope and the precision of the instruments facilitate the identification and manipulation of these structures by the surgeon. In the female pelvis, the gynaecologist can find a dense accumulation of nervous tissue, mainly peripheral nerves and plexuses, which are responsible for the innervation of the pelvic organs and of the muscles of the lower extremities and the pelvic floor. Traumatising these nerves during advanced gynaecological procedures may lead to severe disabilities for the patient after the surgery and should be avoided by the surgeon. Just like any other tissue, the peripheral nerves have their specific structure and organisation and the operator should take utmost care not to damage their fine composition during dissection.

The purpose of this review is to analyse and discuss the current information related to the neurological basis of the pelvic nerves and give some evidence-based recommendations for gynaecological surgeons during neurolysis procedures. We start describing the neuroanatomy of the nerves, their organisations and types of neurolysis. We present the types of nerve injuries and the main repair techniques and results. Finally, we discuss the main findings and draw conclusions. Surgical rules are proposed at the end for safe dissection of the nerves in the different subdivisions of the gynaecological surgery.

## Objective

The present review aims to present and analyse the current information related to the neurological micro-anatomy and neurophysiology of the pelvic nerves and to give recommendations for correct nerve dissection and preservation during gynaecological surgery. The secondary outcome is to expose vulnerable points of pelvic nerves and acquaint the gynaecologist with the basic techniques for neurolysis, applied in other specialties dealing with peripheral nerves.

## Material and methods

Comprehensive research has been done throughout the PubMed database between May 2020 and July 2020 searching for articles and reviews focusing on the following topics: nerve dissection, nerve- sparing techniques and peripheral nerve anatomy. We included 67 studies found under the search of the following MeSH and the keywords terms: Pelvic Nerves AND Neurolysis OR Surgery OR Neuropelveology OR Dissection.

All abstracts were revisited, and the studies were finally selected by two authors (A.A., A.V.) according to the aim of this review.

Initially, a structured investigation question was created using the PICO strategy.

**Structured Investigation Of this Review:** P.I.C.O. Structured Investigation Question

P (Patient/Problem)	Patients undergoing gynaecological or other surgical procedures. Anatomical and physiological studies of pelvic and peripheral nerves
I (Intervention)	Different types of nerve exploration and/or neurolysis required during the surgical procedure or cadaveric lab
C (Comparison)	No comparison
O (Outcome)	Present micro anatomy, neurophysiology, types and common sites of injuries during gynaecological surgeries, repair techniques and neurolysis techniques. Give recommendations for nerve preservation.

Criteria for inclusion were: I. All methodological studies II. English language studies. III. Exploration, dissection, injuries, and repair of pelvic and/or peripheral nerves IV. In vivo and in vitro anatomical nerve articles V: Surgical and cadaveric lab studies

Exclusion criteria include: I. Non-published articles (gray literature). II. Other than English language III. Abstracts which could not be retrieved for review The variables reviewed included epidemiological and clinical data (frequency measures, classifications, clinical scenarios), histopathological and neurophysiological data (neuroanatomy, organisation, neurophysiology, trauma and repair process) and surgical data (exploration techniques, neurolysis techniques, risky gynaecological surgeries)

The sources of information used were digital exclusively, by using PubMed and Google Scholar as search engines.

The primary and secondary data of the study were recorded and tabulated by the authors in the Microsoft Excel program database. Tables and figures were built to present the results obtained. No statistical analysis was planned.

### Peripheral nerves anatomy and organisation

The main neural component of the peripheral nerves are the axons. The axons are projections of the neuronal cells, which conduct electrical impulses between the neurons and the target organs and tissues, or between the neurons themselves. The axons are surrounded by a specific type of glial cells called the schwann cells, which form the characteristic myelin sheath. The main function of the myelin is to protect the axons and to increase the velocity and transmission of the electrical impulses by forming the nodes of Ranvier. Based on the degree of myelinisation and velocity of the signal, the axons are grouped according to the Erlanger-Gasser classification into **type A** fibers (heavily myelinated, high velocity of conduction; abundant in both sensory and motor innervation, including the somatic muscle groups), **type B** (moderate degree of myelinisation and velocity of conduction; present in the visceral afferent fibers) and **type C** fibers (unmyelinated, low velocity of conduction; mainly present in the autonomic nerves).

**Figure 1:**
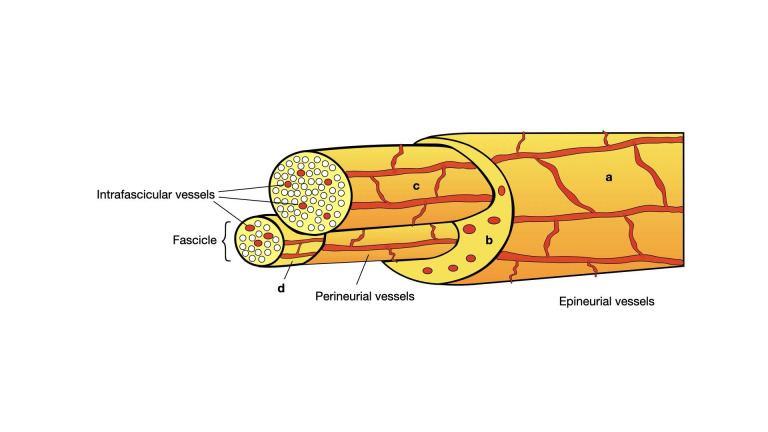
A cross-sectional diagram of a peripheral nerve demonstrating its microanatomy, structural organisation and longitudinal vascular network; a – epifascicular epineurium; b – interfascicular epineurium; c – perineurium; d – endoneurium.

A cross-section of a peripheral nerve shows a specific and well-organised structure created by different types of neuronal connective tissue (Figure 1). The separate axons are grouped into bundles called fascicles. Each fascicle is limited by a thin connective tissue layer called perineurium, while inside the fascicle the individual axons are enveloped by a sheath of endoneurium (Sunderland, 1990). The outermost layer of the nerve and the connective tissue around the different fascicles is called epineurium which, from a microsurgical standpoint, can be further divided into interfascicular epineurium -areolar connective tissue extending between the fascicles, and the epifascicular epineurium – the external sheath that gives the characteristic appearance of the nerve and separates it from adjacent tissues (Millesi et al., 1993; Sunderland, 1990). This division of interfascicular (or internal) and epifascicular (external) epineurium is an important consideration when performing microsurgical operations on the peripheral nerves (Mazal and Millesi, 2005). The loose unspecialised connective tissue surrounding the nerves is defined as paraneurium. Although in tight connection with the peripheral nerves, the paraneurium is not recognised as part of the nerve, which is clearly limited by the epifascicular epineurium (Sunderland, 1990). The dissection at the level of the paraneurium, as long as the epineurium stays intact, is considered safe and is the preferred technique, whenever possible, for nerve dissection (Millesi et al., 1993).

The individual fascicles inside the nerve are not isolated from each other. They are interconnected, constantly splitting and merging together along the nerve length, so that mobilisation of a single fascicule, without causing damage to the rest, with whom they are connected, is impossible (Millesi et al., 1993). The number of fascicles is changing along the course of the nerve. It is considered that the larger the number of fascicles at a particular level, the larger the resistance capacity of the nerve to external compression (Sunderland, 1990). The high number of fascicles at certain areas designates a higher amount of interfascicular epineurium, which allows reorganisation of the fascicular pattern when external compression is applied, without increasing the pressure inside the fascicles (Sunderland, 1990). The fascicular component varies along the nerve, constituting between 30% to 70% of its cross- section, with the rest attributed by the epineurium (Sunderland, 1990). From a gynaecological standpoint, the hypogastric nerve at the level of the sacral promontory consists of 3-21 middle sized fascicles between 0.05-0.2 mm and more than 50 smaller fascicles less than 0.05 mm in diameter,; the former containing between 450-570 nerve fibers and the latter between 40-100 fibers, consisting mainly unmyelinated axons (Jang et al., 2015).

The connective tissue in the structure of the peripheral nerves has a major role for the protection of the nerve fibers from external stress, preventing injuries to the fascicules. The main component, providing tensile strength and elasticity, is the perineurium sheath that defines and organises the individual fascicles (Sunderland, 1990). This layer acts like a diffusion barrier, together with the epineurium, maintaining constant intrafascicular pressure and resisting external compression. The peripheral nerves have a degree of elasticity and capability to stretch, as they are not straight but 10 as undulating along their course. The fascicules, well as the nerve fibers inside the fascicles, also have an undulating pattern (Sunderland, 1990). This way of organisation allows some degree of extension along the nerves when changing the position of the innervated tissues and with movements of the respective organs. When the nerve is pulled and put under traction, the fibers are first strengthened, then stretched and finally rupture if the traction does not cease. According to Haftek (1970), the mean elongation at the limit of elasticity is 69.3% and the point of rupture at 73.3%. The nerve retains its elasticity as long as the perineurium stays intact (Sunderland, 1990). Although mechanically resistant to traction, the vascularisation of the nerve gets impaired, when the nerve is stretched. An elongation of 30% leads to rupture of the epineurial blood vessels, which causes the nerve to become pale (Haftek, 1970). On average the elongation of the nerve of more than 15.7% causes complete arrest of blood flow (Ogata et al., 1986). Another study by Lundborg (1975) shows similar results for cessation of the circulation when stretching to more than 15% of the nerve’s initial length. The discrepancy between the limit for mechanical injury and the impairment in the microcirculation reflects the susceptibility and the precise organisation of the nerves’ vasculature.

The axonal transport and the conduction of electric impulses is provided by the well-organised nerve vascularisation. The nerve vasculature is supplied by regional arteries and veins, originating from vessels in the adjacent tissues (Lundborg, 1979). The largest vessels in the composition of the nerve are arterioles and venules, organised in a longitudinal fashion along the nerve and forming numerous anastomoses along it’s length (Lundborg, 1979). These vessels are positioned in the epineurial connective tissue, both in the epifascicular epineurium and between the fascicles in the interfascicular epineurium. The arteriolar vessels are branching and supply the fascicules in a segmental fashion, so that each epineural arteriolar vessel provides blood for at least 5 fascicules (Lundborg, 1979). At the fascicular level, the vessels are of capillary size and are organised in a longitudinal pattern. A higher concentration of vessels is observed at the level of the perineurial sheath, which gives branches to the endoneurial compartment. The fascicular segmental vascular plexus is the main vascular unit of the peripheral nerves (Lundborg, 1979).

Despite its rich vascular network, the nerve’s microvasculature is susceptible to external damage with traction, elongation, extensive dissection in the adjacent tissues and particularly vulnerable to compression. The external injuries could have a detrimental effect on the nerve function, impairing the normal conduction of signals, and if applied with sufficient force and duration could have long-term effects (Kanaya et al., 1996). Although organised in a segmental fashion, extensive nerve dissection not only decreases the blood flow in the dissected area (Ogata et al., 1986) but could also affects the conduction properties of the whole nerve (Kanaya et al., 1996). Nerve compression is one of the major etiologic factors for peripheral neuropathies and it has been widely examined in different studies. According to Rydevik et al. (1981) compression of 20 to 30 mmHg on the nerve leads to cessation of the venular blood flow. Additional increase of compression between 40 and 50 mmHg leads to cessation of the arteriolar and the intrafascicular circulation. Complete cessation of the blood flow is observed at 60-80 mmHg. The disturbances in the blood supply, leading to venular stasis, can cause an intraneural oedema (Rydevik and Lundborg, 1977), which is considered to be the main pathological mechanism for compressive nerve disorders, like the carpal tunnel syndrome (Sunderland, 1976). The long-term compression leads to perineural fibrosis and interruption of the normal conduction of signals (Starkweather et al., 1978; Millesi et al., 1972). Applying repetitive compression on the nerve could damage its function, leading to prolonged recovery of the conduction of signals and the blood circulation, eventually causing endoneurial oedema (Yoshii et al., 2010). Although it represents only the starting point for further pathological changes, the authors propose that the minor repetitive compression could be one of the risk factors for nerve damage and the breakdown of the blood-brain barrier. A study by Yayama et al. (2010) shows that the congestion due to impairment of the venous flow starts at compression between 0.15 – 0.30 N; further compression of more than 0.30 N impairs the arteriolar flow; complete cessation of the circulation is present at 0.46 N, which corresponds to 48.7 mmHg. It is shown in the same study, the disturbances in the conduction of electrical impulses start even with a lower degree of compression, approximately at 0.2 N, and the compression force of more than 0.3 N leads to complete conduction blockage and intraneural anoxia. For gynaecological surgeons, it is important to emphasise that the force generated between the jaws of the laparoscopic instruments, when grasping tissues is sufficient to completely stop the blood flow in the neural vascular system, as shown by Araki et al. (2017). The study also indicates that the force generated by the grip forceps differs between the different levels of surgical experience – 3 N for the experienced and 7 N for the novice surgeons.

## Techniques for neurolysis

The technique for nerve dissection and neurolysis is well known and is part of the training of plastic, orthopedic and neurosurgeons; however, it is not recognised and implemented in gynaecological practice. The concept of neurolysis was defined by Seddon et al. (1957) as an operation to liberate an injured nerve from scar tissue, fibrosis or other adjacent disease process that interferes with the normal nerve function. The technique was comprehensively described and classified by Millesi et al. (1993) (Table I). Neurolysis begins with exploration and dissection of the nerve from the adjacent structures, releasing it from surrounding adhesions . The surgeon exposes both the proximal and distal unaffected parts of the nerve, where the tissue is completely normal and free of disease. The dissection follows the course of the normal nerve towards the lesion from proximal and distal directions, so that the surgeon can recognise where the lesion starts and from where the neurolysis should begin. The dissection starts from the most superficial layer between the disease (which may involve the paraneurium) and the epifascicular epineurium. If the dissection at this level is sufficient to completely remove the disease from the nerve, it is referred to as external neurolysis. If after excision of the superficial disease the infiltration expands deep into the nerve and reaches beyond the epifascicular epineurium, internal neurolysis may be necessary. At this moment the surgeon must consider the risks and benefits of more extensive dissection. Internal neurolysis may severely disrupt the normal function and organisation of the fascicles as well as the vascularisation, potentially causing more harm than the disease itself (Mazal and Millesi, 2005). As previously described, the fascicles inside the nerve are interconnected and dissecting even a single fascicle could impair the whole nerve structure (Sunderland, 1990). Internal neurolysis consists of a few separate steps. The first step is epifascicular epineurotomy, which consists of one of few longitudinal incisions across the disease thickened portion of the nerve. The compressed fascicles could start expanding from the incision site. If after this epineurotomy the nerve continues to look deformed, the surgeon must remove the epifascicular epineurium around the circumference of the nerve in the diseased area so that the fascicles can be released: this is an epifascicular epineurectomy. If after excising the epineurium, fascicles are still involved and compressed by the disease, an interfascicular epineurectomy is performed. At this stage, severe damage may be caused by the surgeon, as in his attempts to be exhaustive and remove all the fibrotic tissue, the interfascicular connections and circulation may be injured. The internal neurolysis must stop immediately after decompression of the nerve is achieved (Millesi et al., 1993).

**Table I:** Types of neurolysis (Millesi et al.,1993; Mazal and Millesi, 2005).

Type of neurolysis	Description
Exploration	Dissection around the nerve Exposure of normal/undamaged segments of the nerve proximal and distal to the lesion
External neurolysis	The nerve is liberated from all the adhesions in the paraneurial space Fibrotic tissue exerting compression on the nerve are excised The epifascicular epineurium stays intact
Epifascicular epinerotomy	Incisions are made on the thickened part of the nerve where fibrotic tissue is still present If there are signs of decompression (fascicles start to spurt out from the incision sites) the procedure is complete
Epifascicular epineurectomy	Epifascicular epineurium is excised all around the nerve in attempt to decompress the fascicles distally from the epineurotomy
Interfascicular epineurectomy	Fibrotic tissue is removed between the fascicles The procedure is limited only to the segment and fascicles affected by the fibrosis. The organisation between the non-affected fascicles should not be disrupted in any case

In gynaecological practice, management of severe deep endometriosis may require the surgeon to perform neurolysis, as in some cases the endometriotic lesions can envelope and even infiltrate the pelvic nerves (Possover and Chiantera., 2007). When performing external neurolysis, the surgeon must be precise with the dissection of the nerves and take utmost care not to cause additional trauma of the neural structures. The surgeon must take his time to find the exact plane between the epineurium and the adjacent tissues using fine and precise instrumentation. The characteristics of the instruments and the source and type of energy used are important factors to prevent nerve damage. In maxillofacial surgery, the atraumatic dissection of the facial nerve is a key step in parotidectomy. When dissecting the nerve from the adjacent structures, sharp dissection only with cold scissors is the recommended approach (Graf and Petruzelli, 2018). Using electric or ultrasonic energy close to the nerve could potentially lead to thermal damage, especially using an ultrasonic device, as it is associated with prolonged active blade hyperthermia (Bandi et al., 2008). In his article concerning facial nerve dissection, Sachs and Conley (1981) describes exhaustively his technique for neurolysis. The author uses small blunt cold scissors of Stevens type. The surgeon finds the dissection plane between the epineurium and the surrounding tissues and enters the plane with closed scissors. The belly of the scissors is oriented towards the nerve and the blunted tip faces the surrounding structures. The surgeon slides the scissors in a closed fashion along the nerve, separating it from the surrounding tissues. After a certain distance, which depends on the extent of the fibrosis and characteristics of the tissues, the scissors are carefully opened. The surgeon repeats all these steps until complete dissection of the nerve. The blunt tip, the orientation of the scissors in relation to the nerve and the dissection using the embryological planes prevents undesirable neural damage. The author emphasises the delicacy of this technique with an associated reduction in nerve trauma and subsequent decrease in postoperative complications.

## Classification of nerve injuries

Two classifications for nerve injuries are widely recognized. The first one was defined by Seddon (1943) and includes three types of injuries: neuropraxia, axonotmesis and neurotmesis. Later, in 1951 Sunderland further developed the classification by dividing the neurotmesis into three different degrees of injuries, totaling 5 classes of nerve lesions (Sunderland, 1951). Each type of injury represents a specific pathomorphological lesion affecting the nerve and its connective tissue (Table II).

**Table II:** Classification of nerve injuries (Seddon et al., 1943; Sunderland, 1951).

Degree of injury	Characteristic
Neuropraxia (type I)	Focal segmental demyelinisation Conduction blockage Mild sensory/motor disturbances No wallerian degeneration Complete and spontaneous recovery
Axonotmesis (type II)	Axonal injury with preserved endoneurium Wallerian degeneration is present Sensory and motor deficits are present Normally a complete recovery follows; in some situations, surgery may be necessary
Neurotmesis (type III)	Axons are damaged together with the endoneurial sheath The perineurium is intact and the fascicular structure is preserved Sensory and motor deficits are present Surgical intervention is usually necessary
Neurotmesis (type IV)	The perineurium is damaged and the fascicular pattern is disorganised Only the epineurium is preserved from the neural connective tissue Severe and motor deficits Surgical intervention is necessary
Neurotmesis (type V)	All the connective tissue layers of the nerve are damaged The normal structure and continuity of the nerve is lost Severe sensory and motor deficits Surgical intervention is necessary

Neuropraxia (type I injury according to Sunderland) is the mildest form of nerve injury. It represents a temporary conduction block with preserved axonal continuity at a specific area of the nerve. The conduction of nerve signals is preserved proximally and distally from the injured segment, but it is blocked across it (Sunderland, 1990). The connective tissues in the structure of the nerve (endo-, peri-, epineurium) are preserved. As the axons are damaged, but not destroyed, there is no wallerian degeneration. Clinically, there are mild sensory or motor disturbances below the level of injury. Usually there is full regeneration and recovery of function a few days to weeks after the injury.

Axonotmesis (type II injury according to Sunderland) represents a discontinuation of the axon, but with fully preserved connective tissue. The axonal damage is severe and distally from the lesion wallerian degeneration occurs. The conduction distally from the lesion is completely blocked and sensory and motor deficits are present. The architecture of the nerve, organised by the connective tissue, is preserved and the axons regenerate along the same sheath of endoneurium that contained the nerve fibers before the injury (Sunderland, 1990). The complete recovery takes from weeks to months, but in some cases, it might be disturbed by fibrosis of the adjacent tissues, damaged by the same injury.

Neurotmesis (types III, IV and V injuries according to Sunderland) is the most severe form of nerve injury and involves not only the axons but also the surrounding connective tissue, which outlines the structure of the nerve. Neurotmesis, as a term defined by Seddon, is characterised by damage of the neuronal connective tissue in general, while the different degrees of injury according to Sunderland correspond to damage on the separate types of connective tissue (endo-, peri-, epineurium). In type III injury the axon is damaged together with the endoneurial sheath, while the perineurium and the epineurium stay intact. The fascicular pattern is preserved, but the internal organisation of the fascicles is distorted. In type IV injury the perineurial fascicular sheath is disintegrated and in type V injury the whole nerve is completely transected, with the epineurium being impaired. In neurotmesis, the continuity of the nerve is lost, and severe sensory, motor and autonomic disturbances are present distally from the injury site.

When injured intraoperatively, the peripheral nerves may be repaired by the surgeon using a microsurgical technique. In cases of sharp transection with clear lines, an end-to-end anastomosis may be achieved. When the nerve is injured on a larger scale, the damaged part is excised; the proximal and distal nerve ends are connected either using an end-to-end anastomosis or using a nerve graft. Regardless of the method, it is of paramount importance to keep the nerve and the anastomosis tension free, otherwise the blood flow and respectively the healing process, will be impaired (Clark et al., 1992). The end-to-end anastomosis may be completed by performing an epineurial repair or even a perineurial repair, connecting the individual fascicles in a larger nerve. One of the drawbacks with the epineurial repair is the malalignment of the fascicles and the loss of function postoperatively despite precise intraoperative technique. In the female pelvis the autonomic nerve fibers are too delicate to be repaired and injuring them can lead to loss of their function that may eventually be overtaken by patent nerves on the contralateral side. During gynaecological surgeries the most commonly injured somatic nerve is the obturator nerve, encountered during pelvic lymphadenectomy. In most articles regarding obturator nerve injury, the nerve is sharply dissected and repaired using an end-to-end epineurial anastomosis with a 6-0 nonabsorbable polyester suture (Ricciardi et al., 2012). Another option in large injuries is to use the sural nerve as a nerve graft (Harma et al., 2014; Dias et al., 2014). Most of these articles demonstrate excellent postoperative results with absent or minor motor and sensory deficits when immediate repair is performed, followed by an appropriate combination of physiotherapy and neurotropic medications. According to other studies nerve repair cannot lead to the same functional capacity as before the surgery due to fibrosis and scarring of the perineurium (Wang et al., 2019). Nevertheless, intraoperative repair of the obturator nerve immediately after its recognition is an effort that should be considered to best restore its function.

**Table III:** Types of nerve repair techniques (Vasilev, 1994).

Type of repair	Characteristic
Epineurial repair	Anastomosis of the most superficial sheath of the nerve Appropriate for medium-size nerves without many fascicles Nonabsorbable 6-0 polyester suture Method of choice for obturator nerve injury Possible misalignment of the individual fascicles
Perineurial repair	Anastomosis of the perineurial sheath of the individual fascicles Better alignment of the fascicles Appropriate for large-size nerves
Graft repair	Should be applied to achieve a tension-free anastomosis Sural nerve can be used as a graft

## Discussion

Nerve injury following advanced gynaecological procedures may lead to significant postoperative morbidity, therefore the concept of nerve-sparing procedures has been developed and has gained significant popularity in the recent years (Muallem et al., 2019; Darwish and Roman., 2017; Ercoli et al., 2017; Seracchioli et al., 2019). Nerve preservation includes dissecting the nerves and lateralising them from the field of action, but also correct handling and manipulation, taking into consideration their delicate structure and organisation. In this sense, the minimally invasive approach has a clear advantage over open surgery. This is due to the anatomical magnification and fine instrumentation, allowing precise surgical dissection and distinction between different structures, specifically the vessels and nerves. During gynaecological procedures, one can encounter structures both from the autonomic nervous system (the superior and inferior hypogastric plexuses, the hypogastric nerves and the pelvic splanchnic nerves) and from the somatic nervous system. The most commonly encountered somatic nerves are the obturator and genitofemoral nerves, but also the sciatic, the pudendal, the lumbosacral trunk and the sacral plexus in cases of deep pelvic dissections. Whilst a degree of elasticity and the capacity to resist compression maintains constant intrafascicular pressure, unnecessary manipulation and grasping of the nerves should be avoided. As it was emphasised, the force generated between the jaws of the grasping forceps is sufficient to cease the circulation in the intrafascicular vascular system. The nerves should not be pulled, and traction should not be exerted, especially over a long period of time, as this may impair the blood supply. Moving the uterus from side to side after cannulation is usually sufficient to expose the hypogastric nerves, without grasping or manipulating them. It is well known that unnecessary and extensive ureterolysis should be avoided, especially in deep endometriosis surgery, as this could damage the adventitia of the ureter and lead to ureteral fistula (Uccella et al., 2014). The same concept should be acquired for the nerves. Wide dissection and exposure of the nervous structures inevitably leads to impairment of the vascularisation in the area and eventually could cause scar tissue formation and fibrosis, which in turn could become a factor for postoperative pelvic pain. As described by Seracchioli et al. (2019), during advanced pelvic surgeries with wide retroperitoneal dissection, the intrafascial approach should be applied, based on the Toldt’s law of fascial coalescence (Toldt et al., 1927). Acquiring this technique allows the surgeon to stay in the safe area between the fascia propria of the rectum and the hypogastric fascia. The surgeon, working in this space, remains ventral to the hypogastric nerve and the superior hypogastric plexus, without performing unnecessary dissection to expose them.

The gynaecological surgeon encounters different nervous structures depending on the nature of the disease that he or she is managing and the respective operative area in the pelvis. In gynaecological oncology, the deep uterine vein is recognised as an important landmark, caudal to which are the delicate fibers of the pelvic splanchnic nerves. The surgeon must respect the veins’ anatomical position and stay cranial to it when performing a nerve-sparing procedure (Muallem et al., 2019; Raspagliesi et al., 2004). By developing the Okabayashi’s space, the surgeon lateralises the hypogastric nerves, distancing them from the uterosacral ligaments. Special attention should be paid to the obturator nerve during the lymph node dissection in the paravesical fossa and in the lumbosacral space, as well as the genitofemoral nerve at the level of the psoas muscle.

In prolapse surgery and particularly in sacrocolpopexy, when dissecting the promontory, attention should be paid not to lacerate the median sacral vessels. If cut this could lead to unnecessary bleeding and coagulation, causing thermal trauma to the hypogastric plexus. The latter should be gently lateralised by the surgeon, without unnecessary manipulation or traction. The same rules apply for the hypogastric nerve, which is visualised and lateralised from the field of dissection, when preparing the area in the pararectal fossa for placement of prosthesis (Ercoli et al., 2017). Alongside the nerve, the surgeon should also be careful not damage the right ureter, as it crosses the same area. The ureter itself may be used as a landmark for the identification of the hypogastric nerve, as shown by Seracchioli et al. (2019). The hypogastric nerve is an important nervous structure carrying the sympathetic fibers to the inferior hypogastric plexus; it should always be recognised and preserved in this type of surgery.

In deep endometriosis surgery, the nodule may be positioned in such a way that it infiltrates the neural structures situated in the pelvis. When the thin fibers of the autonomic nervous system are involved, including the pelvic splanchnic nerves and inferior hypogastric plexus, sparing these nerves from damage is largely impossible despite precise dissection techniques or a highly experienced surgeon (Landi et al., 2009; Darwish and Roman., 2016). As stated by Possover et al. (2007b), nerve- sparing may be accomplished by protecting neural structures on the uninvolved side from injury. In complex cases, when both sides are involved, the surgeon may perform complete resection on the side more aggressively involved by the disease, as in such cases the endometriosis has already irreversibly damaged the nerve fibers. They may leave some disease on the other side of the pelvis in order to preserve the autonomic innervation (Darwish and Roman, 2016).

Postoperative chronic pelvic pain due to retroperitoneal fibrosis and nerve entrapment is a well-recognised concern in complex gynaecological surgeries. Occasionally, despite the complete excision of the disease, the postoperative adhesions and fibrosis continue to disturb the patient’s quality of life and necessitate additional treatment. This issue is particularly concerning in cases of endometriosis, when the adhesion formation can reach up to 90% of the cases (Canis et al., 1992). Prevention of both adhesions formation and fibrosis should be among the primary goals of the surgeon when managing the disease. Currently, the most reliable method for prevention of postoperative fibrosis is by applying the rules of microsurgery, defined by Gomel: delicate tissue handling, judicious use of energy, intermittent irrigation of the operative field, meticulous hemostasis and dissection in the correct surgical planes (Gomel and Koninckx et al., 2016). Low-intra abdominal pressure, humidified CO2 and perioperative corticosteroids have also proven to minimise peritoneal trauma and fibrosis and decrease postoperative pain (Querleu et al., 1989; Matsuzaki et al., 2011; Canis et al., 2018). According to the Cochrane meta-analyses, the commercially available barrier agents for adhesion prevention have not proven any benefit in terms of postoperative pain or live birth rate, such that that the surgeon should not rely on these products and should instead use a microsurgical technique (Ahmad et al., 2020).

The pelvic autonomic nerves are particularly fragile structures and dissecting them as part of advanced gynaecological procedures may lead to damage to the fibers. In these cases, the surgeon should avoid unnecessary manipulation and, if it is feasible, to apply a “non-touch dissection” on the nerves, exerting no mechanical force with the instruments that could damage the fibers. An device that could be used for secure nerve dissection is the cavitron ultrasonic surgical aspirator (CUSA). The ultrasonic energy of this device aspirates the cells with high water content (like adipose tissue) without damaging the surrounding tissues rich in collagen, and hence used for liposuction (Hao et al., 2016). Currently CUSA is applied in different fields of medicine including liver and spleen resection, neurosurgery and plastic surgery. In their pilot study Hao et al. demonstrate the advantage of this system for nerve dissection in nerve- sparing radical hysterectomy, showing more rapid recovery of voiding function and shorter hospital stay in the CUSA group compared to the control (Hao et al., 2016). The patients in the CUSA group also had significantly less blood loss, with the authors concluding that the nerve-sparing radical hysterectomy using CUSA is safe, feasible and preserves the autonomic innervation.

Another encouraging method that could be used for non-touch nerve dissection is by using a water- jet stream. This technique is already implemented in neurosurgery and hepato-biliary surgery for dissection of parenchymal organs (Piek et al., 2002; Kauff et al., 20162). The advantages of this device are preservation of the blood vessels, reduced blood loss and parenchymal trauma and practically no thermal damage (Schurr et al., 1994; Piek et al., 2002). In a study by Tschan et al. (2010) the authors examine the potential advantages of the water-jet dissection for peripheral nerves neurolysis. (Tschan et al., 2010). The results from the study show that the integrity of the sciatic nerve in experimental rats remained preserved with a water-jet pressure of up to 30 bar . Increasing the pressure of the water- jet above 40 bar led to structural and functional damage, which resolves completely after 12 weeks, as shown by the histological studies. The authors concluded that water-jet may be safely applied up to pressure of 30 bar in clinical conditions and is suited for dissection of the peripheral nerves from the adjacent fibrotic tissues. In the current literature there are only a few articles studying the potential benefit of water-jet application in gynaecology, particularly in nerve– sparing radical hysterectomy (Li et al., 2019; Li et al., 2019; Wu et al., 2016). These studies show promising results, as the water-jet allows more precise dissection of the nerve fibers, which facilitates their identification and protection by the surgeon. With the water-jet stream the surgeon can safely dissect the individual fibers, without causing any significant damage that would compromise their function. In these studies the patients in the water-jet groups displayed better urodynamic results and a shorter recovery time to normal micturition compared to the control groups, without compromising the survival rates. The water- jet dissection is an interesting technique that needs wider application in the field of gynaecology to demonstrate its advantages for nerve dissection.

**Table IV:** Common gynaecological procedures with their corresponding vulnerable sites for nerve injuries.

Gynaecological field/ procedure	Sites of nerve injury
Oncology - Pelvic lymphadenectomy - Radical hysterectomy	Genitofemoral nerve – lying on the psoas muscle; in close proximity to the external iliac lymph nodes Obturator nerve – in the paravesical fossa; important landmark during lymphadenectomy Hypogastric nerve – dissected from the uterosacral ligaments by developing the Okabayashi’s space when preparing the posterior parametrium Pelvic splanchnic nerve – below the level of the deep uterine vein; important landmark when developing the lateral parametrium
Pelvic organ prolapse - Sacrocolpopexy	Superior hypogastric plexus – at the level of the promontory Hypogastric nerve – at the level of the promontory and during the pararectal dissection
Deep endometriosis	Could involve any of the pelvic nerves Most common sites – the pelvic splanchnic nerves, the inferior hypogastric plexus, the sacral roots Meticulous dissection should be done with sparing of the uninvolved nervous tissue In case of unilateral disease, the contralateral site should be preserved In case of bilateral disease, the site which is more symptomatic/ with more aggressive infiltration should be excised with preservation of the contralateral nerves (in case of bilateral resection – risk of neurological deficits) Postoperative retroperitoneal fibrosis causing chronic pelvic pain is a major concern – microsurgical rules with means of adhesion prophylaxis should be applied

In order to be spared, the nerves should first be recognised. Possover and his team have developed a technique for intraoperative nerve dissection and identification called the laparoscopic neuro- navigation technique, or LANN. The procedure consists of stimulation of the different nerve fibers, after their dissection and exposure, using a bipolar forceps with specific characteristics of the electric current (square-wave pulse duration of 250 μs, pulse frequency of 35 Hz and electric potential of 12 V (Possover et al., 2007a; Possover et al., 2007b). A rectal probe and a dual sensor transurethral catheter are placed in the rectum and in the bladder respectively to indicate pressure changes caused by the muscle contractions caused by the electrical stimulation. The principle of neuromapping using electric stimulation has also been applied in rectal surgery (Kneist et al., 2014). An ongoing clinical trial named NEUROS, stated as the first randomised control trial comparing the functional outcome in rectal cancer patients undergoing total mesorectal excision (TME) with and without pelvic intraoperative neuromonitoring, will show us the real advantages of neuronavigation in advanced pelvic operations (Kauff et al., 2016). In the field of gynaecology, the laparoscopic neuronavigation has been proven as an effective way of reducing the postoperative morbidity and preservation of bladder function and it is reasonable to recommend it in pelvic surgeries with increased risk for nerve damage (Possover et al., 2005).

Development of a nerve-specific agent which could facilitate the identification of the nervous structures has been the objective in many studies from various medical specialties (Walsh et al., 2019). The aim in these studies is to find a fluorescent dye with affinity to the nervous tissue that, when stimulated with light from the near- infrared spectrum, emits fluorescent light that may be detected with specifically designed cameras and aid the recognition of the nerves by the surgeon (Gibbs, 2012). Different agents have been used in animal models (Oxazine 4, BMB, GE 3126, etc.) but until now the only two fluorophores approved by the American Food and Drugs Association (FDA) are the methylene blue and the indocyanine green (ICG) (Walsh et al., 2019; Jo and Hyun., 2017). Currently there are few studies examining the effects of ICG for nerve identification, based on the illumination of the vasa nervorum after intravenous injection of the dye. Indocyanine green has been used in radical prostatectomy, for visualisation of the facial nerve, the phrenic nerve and the thoracic sympathetic system (Mangano et al., 2018; Wagner et al., 2012; Chen et al., 2015; He et al., 2018). Unfortunately, the ICG is not a nerve-specific agent and it loses specificity in highly vascular areas (Walsh et al., 2019). Further studies are needed to evaluate the real advantage of the ICG for the identification of peripheral nerves.

In cases when the somatic nerves are involved by fibrosis, as with endometriotic nodules, the surgeon must follow the main principles for neurolysis, defined by Millesi (Millesi et al., 1993; Mazal and , Millesi, 2005; Millesi, 1979). As nerve dissection and repair techniques are usually not part of standard gynaecological training, it should be emphasised that our recommendations for neurolysis in pelvic surgeries are taken from other specialties and modified in such a way that gynecologists may implement them in their practice. The dissection should preserve the integrity of the nerve as much as possible and should not itself cause additional trauma. As recommended by Graf and Petruzelli (2018)., simple instruments like cold scissors should be the preferred tools, as using other more sophisticated devices with electric or ultrasonic energy could cause thermal damage to the nerve. The nerves should be dissected proximally and distal from the site of the lesion, so that the surgeon is confident that all the diseased area is exposed and completely removed. The somatic nerves are located deep in the pelvis and so their primary dissection during the first operation could be complex and high risk, even in experienced hands. The surgeon must be as complete and prudent as possible during the first surgery, as with each subsequent operation, the degree of surgical challenge and risk increases. The neurolysis must be limited to the area of the nerve infiltrated by the disease and should not involve the unaffected part. The excision of the nodule should be performed superficially on the nerve, without damaging the epifascicular epineurium, with externalwith external neurolysis being the preferred technique. In cases when the endometriosis infiltrates deep in the nerve, epifascicular epineurectomy may be performed, together with excision of the fibrosis affecting the interfascicular epineurium. Performing the procedure with thinner laparoscopic instruments of 3 mm instead of the conventional 5 mm is preferred in this type of intrafascicular dissection. These kinds of instruments have been successfully applied in different specialties, including gynaecology (Ghezzi et al., 2008; Breda et al., 2015). Using the fine caliber tips of these instruments facilitates the excision of the lesion with maximum preservation of the surrounding fascicles. A study by Béguinot et al. (2020) has shown that the main benefit from utilisation of 3 mm instruments is the cosmetic appearance for the patient at the expense of longer operative time and deterioration of the surgeons working conditions. (Beguinot et al., 2020). In theory, using 3 mm instruments could lead to more precise excision, preservation of the separate fascicles and decreased postoperative morbidity, but considering the complexity of these types of surgeries and their duration further studies are needed to conclude if the use of fine-caliber instruments is of overall benefit to the patient. Nevertheless, the surgeon should keep in mind that the excision of the intrafascicular epineurium will certainly damage the nerve fascicles and will lead to neurological impairment to some degree. In his follow-up study of patients with sciatic nerve endometriosis, some of which who had undergone more than 30% excision of the nerve, Possover shows encouraging results in terms of reduction of pain. It also showed that at least three years of intensive physiotherapy may be necessary to recover the normal gait after the surgery (Possover, 2017). It must be underlined that these kinds of surgeries must be performed in specialised medical centers, dedicated to deep endometriosis surgery, by an expert surgeon with comprehensive knowledge in the field of neuropelveology.

In conclusion, the nerves have a complicated and precise anatomical structure that must be respected at all times by gynaecological surgeons. Unnecessary manipulation on the nerves should be avoided with nerve-sparing techniques being the preferred procedure. Whenever possible, in cases where nerve dissection is necessary, the main principles of microsurgery and neurolysis should be respected.
